# Influence of augmented reality technique on the accuracy of autotransplanted teeth in surgically created sockets

**DOI:** 10.1186/s12903-024-04173-1

**Published:** 2024-04-05

**Authors:** María Teresa Marhuenda Ramos, Ignacio Faus-Matoses, Álvaro Zubizarreta-Macho, Elena Riad Deglow, Ana Belén Lobo Galindo, Francesc Abella Sans, Alba Belanche Monterde, Vicente Faus Matoses

**Affiliations:** 1 Department of Stomatology, Faculty of Medicine and Dentistry, University of Valencia, 46010 Valencia, Spain; 2 Department of Dentistry, Faculty of Health Sciences, Alfonso X El Sabio University, 28691 Madrid, Spain; 3 Department of Surgery, Faculty of Medicine and Dentistry, University of Salamanca, 37008 Salamanca, Spain; 4 Department of Endodontics, Universitat Internacional de Catalunya, 08195 Barcelona, Spain

**Keywords:** Accuracy, Augmented reality, Cone-beam computed tomography scan, Digital impression, Tooth autotransplantation

## Abstract

**Background:**

The objective of the present study was to evaluate the reliability of an augmented reality drilling approach and a freehand drilling technique for the autotransplantation of single-rooted teeth.

**Materials and methods:**

Forty samples were assigned to the following surgical techniques for drilling guidance of the artificial sockets: A. augmented reality technique (AR) (n = 20) and B. conventional free-hand technique (FT) (n = 20). Then, two models with 10 teeth each were submitted to a preoperative cone-beam computed tomography (CBCT) scan and a digital impression by a 3D intraoral scan. Afterwards, the autotrasplanted teeth were planned in a 3D dental implant planning software and transferred to the augmented reality device. Then, a postoperative CBCT scan was performed. Data sets from postoperative CBCT scans were aligned to the planning in the 3D implant planning software to analize the coronal, apical and angular deviations. Student’s t-test and Mann–Whitney non-parametric statistical analysis were used to analyze the results.

**Results:**

No statistically significant differences were shown at coronal (*p* = 0.123) and angular (*p* = 0.340) level; however, apical deviations between AR and FT study groups (*p* = 0.008) were statistically significant different.

**Conclusion:**

The augmented reality appliance provides higher accuracy in the positioning of single-root autotransplanted teeth compared to the conventional free-hand technique.

## Introduction

Tooth autotransplantation consists of the substitution of an autogenous tooth on the place of another tooth that cannot be conserved. It is carried out by the extraction of the donor tooth and its placement inside the recipient socket previously prepared. The third molars, premolars, canines and supernumerary teeth have been used as donor teeth for autotransplantation. The reasons why many surgeons do not indicate autotransplatations are the variability in this procedure success rate data and the widely use of titanium implant rehabilitations. However, the autotransplanted teeth have some advantages than implants such us the maintenance of the proprioception of the periodontal ligament, conservation of the natural aesthetics, the possibility of performing orthodontic movements [1] and the preservation of the alveolar bone and gingiva. Also, autotransplantation can be indicated in growing patients [2]. The procedure of autotransplantation consists on the extraction of the not conserved tooth and the correct preparation of the recipient site with handpiece. After that, the donor tooth is carefully extracted with forceps and profuse irrigation in order to maintain the periodontal cells and cementum. Then, the donor tooth is immediately placed in the recipient socket and it is initially fixed by a retainer to the adjacent teeth [3]. The correct stabilization of the autotransplanted tooth and the selection of an appropriate recipient socket are critical consideration for the success of this treatment [4]. In addition, has been reported success rates of autotransplantation between 30 to 100% in one to five years. The ankyloses rates are proximately of 2% [5]. Whereas, success rate can depend on the maturation of the donor tooth because the immature teeth have the capability of maintain its vitality after transplantation [6]. The surgical procedure has been modified and the construction of three dimension (3D) replica of the donor tooth has been used to increase the precision of the socket preparation. Also, orthodontic movement after the transplantation has been used to prevent ankyloses and regenerate the periodontium [7]. The sterolithographic replicas of donor tooth are printed from an initial cone beam computer tomography (CBCT). This can reduce the surgical time and increase the success rate of autotransplantation. Moreover, 3D guides have been indicated in order to properly positioning the donor tooth in the socket and avoiding the injury of the donor ligament and the adjacent teeth [8]. The postoperative position of autotransplanted teeth has been studied by comparing the 3D preoperative planning and the 3D final position of the tooth. The tooth can also be positioned with assisted by computer with static navigation technique. Raid-Deglow et al. obtained significant differences in the apical position of the autotransplanted tooth when it was positioned with static navigation technique (ST) (5.65 ± 2.81 mm) than with free-hand positioning (FT) (3.90 ± 1.99 mm) [9]. ST has showed satisfactory results in other fields of dentistry and it is a precise and accurate method of positioning autotransplanted teeth allowing to change the direction of colocation in real time [9] Also, another study showed angular deviation between the planned and the final position of 5.6 ± 5.4° and apical deviation of 2.61 ± 0.78 mm using 3D surgical splints [10]. However, planning guides reduce the surgical complications and avoid an invasive osteotomy of the recipient alveolar bone [11]. The improvement in the surgical procedure of autotransplantation is a promising fact to increase the success and the indication of this procedure [4, 5, 11].

The objective of the present study was to evaluate the reliability of an augmented reality drilling approach and a freehand (FT) drilling technique for the autotransplantation of single-rooted teeth. The null hypothesis (H_0_) stated that the AR and FT surgical techniques for drilling guidance of the artificial sockets did not show differences for the autotransplantation of single-rooted teeth.

## Materials and methods

### Study design

**Fig. 1:**
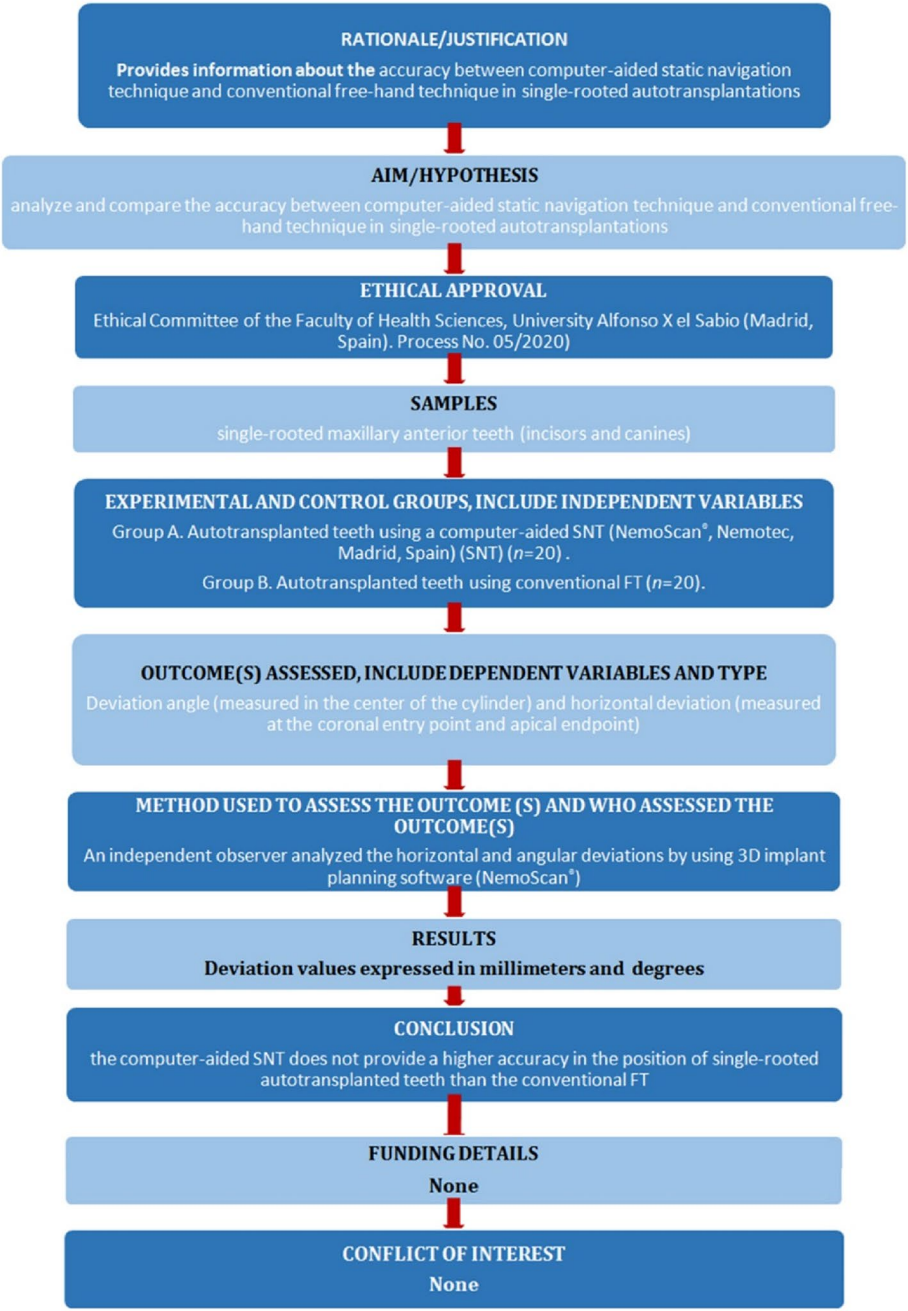
Preferred reporting items for laboratory studies in endodontology flowchart

Forty single-rooted maxillary anterior teeth (incisors and canines), extracted for periodontal or orthodontic reasons, were selected for this study conducted at the Dental Centre of Innovation and Advanced Specialties at Alfonso X El Sabio University (Madrid, Spain) between January and March 2022. The sample size was selected according to a previous study with a power effect of 88.4 (it is considered acceptable from 80) [9]. The manuscript of this laboratory study has been written according to 2021 Preferred Reporting Items for Laboratory studies in Endodontology (PRILE) guidelines (Fig. 1) [12, 13]. In addition, the study was conducted in accordance with the principles defined in the German Ethics Committee’s statement for the use of organic tissues in medical research (Zentrale Ethikkommission, 2003), the Declaration of Helsinki and was authorized by the Ethical Committee of the Faculty of Health Sciences, University Alfonso X el Sabio (Madrid, Spain), in October 2020 (Process No. 05/2020). All the patients signed an informed consent form to donate the teeth for the present study.

### Experimental procedure

The single-rooted teeth were mounted into two experimental epoxy resin models (Ref. 20–8130-128, EpoxiCure®, Buehler, IL, USA), each with 20 teeth. Ten teeth (for autotransplantation) were placed in the internal part of the model, and 10 teeth (used as a reference to align the virtual digital file to the real scene), in the external part. The teeth were randomly (Epidat 4.1, Galicia, Spain) assigned to two study groups: Group A, augmented reality appliance (Hololens2, Redmond, WA, USA) (AR) (*n* = 20), and Group B, autotransplanted teeth using conventional free-hand technique (FT) (*n* = 20). All experimental and measurement procedures were based on a previous study of Riad-Deglow et al. [9].

**Fig. 2:**
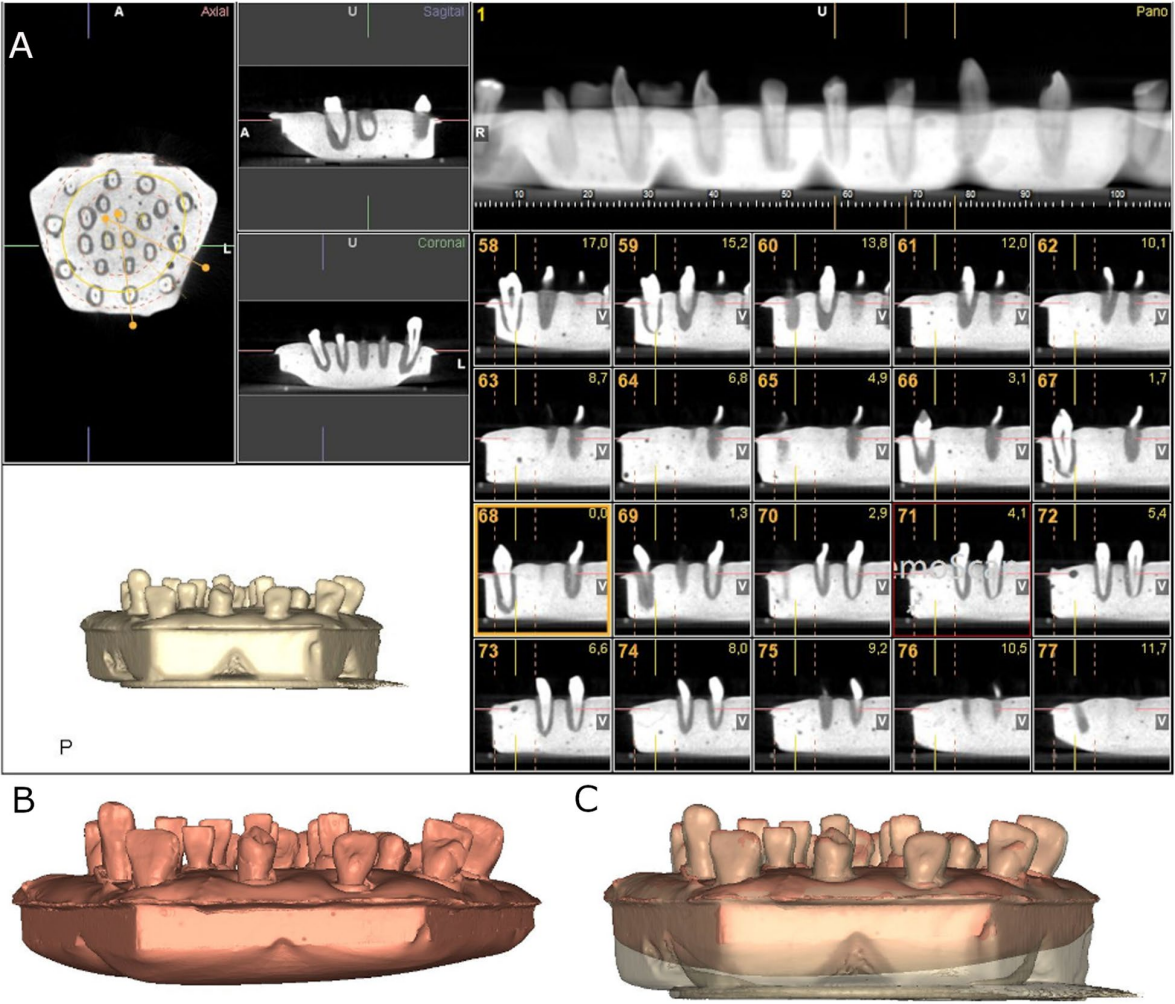
**A** CBCT scan, **B** STL digital files and **C** alignment of the digital workflow

The two experimental models were submitted to a preoperative cone-beam computed tomography (CBCT) scan (WhiteFox, Acteón Médico-Dental Ibérica S.A.U.-Satelec, Merignac, France) with the following exposure parameters: 105.0 kilovolt peak, 8.0 milliamperes, 7.20 s, and a field of view of 15 × 13 mm (Fig. 2A). Subsequently, a digital impression was made using a 3D intraoral scan (True Definition, 3 M ESPE™, Saint Paul, MN, USA) by means of 3D in-motion video imaging technology to generate a standard tessellation language (STL) digital file (Fig. 2B). The 3D intraoral scan (True Definition) uses a cloud of points that create a tessella network, representing 3D objects as polygons composed of equilateral triangle tessellas [14, 15]. The image capture procedure was performed by scanning the palatine and occlusal surface followed by the buccal surface, according to the manufacturer’s recommendations. Datasets obtained from this digital workflow were uploaded to a 3D implant planning software (NemoScan®) to plan the placement of autotransplantation in Group A (Fig. 2C).

**Fig. 3:**
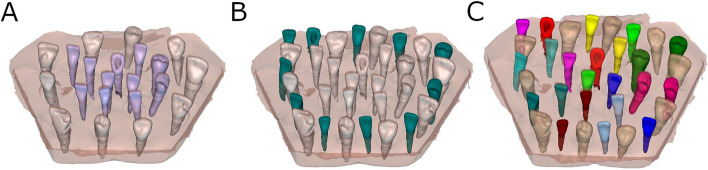
**A** Frontal view of the segmented teeth selected for autotransplantation (purple teeth), **B** frontal view of the segmented teeth selected for autotransplantationplaced on the planned position (green teeth) and **C** descriptive illustration of the position of each autotransplanted tooth

After matching the 3D surface scan and CBCT data (WhiteFox), each tooth in the internal part of the model was individually segmented and virtually placed between the teeth placed outside of the model (Fig. 3).

Then, the virtually autotransplanted teeth were downloaded as a STL digital file from the 3D implant planning software (NemoScan®). Afterwards, the multi-platform augmented reality and augmented reality application development platform (Version 6.5.22, Vuforia, Unity Technologies) was installed in an augmented reality appliance (Hololens2, Redmond, WA, USA) for experimental model tracking in real-time. Furthermore, the recognition and subsequently alignment process between the virtual image and the experimental model was accomplished through Vuforia's proprietary feature detection algorithms, which was programmed to search for reference anatomical features such as corners, edges or points where there is a difference in curvature, such as dental cusps. Finally, this STL digital file was loaded in the augmented reality application (DentalGlasses, beta version) to visualize the STL digital file on the orography of the experimental model.

The surgically created sockets of the teeth were randomly assigned to the AR study group; the drilling was performed by means of an augmented reality appliance (Hololens2, Redmond, WA, USA), to allow the visualization of the endodontic access cavities in all space planes. Specifically, the mixed reality eyepiece identified, interpreted, and reacted to embedded voice commands (keywords) as well as predetermined hand signs, enabling holographic navigation of the app (DentalGlasses, beta version) installed on the AR eyepiece. After this, the AR eyepiece performed digital spatial mapping of the physical environment using 4 visible light cameras, 2 infrared cameras, a 1 MP time-of-flight depth sensor, an accelerometer, a gyroscope, and a magnetometer arranged on both sides of the MR optical device, which allowed the recognition and monitoring of the artificial epoxy resin models with the teeth embedded in real time as well as the alignment of the holographic digital content on them.

**Fig. 4:**
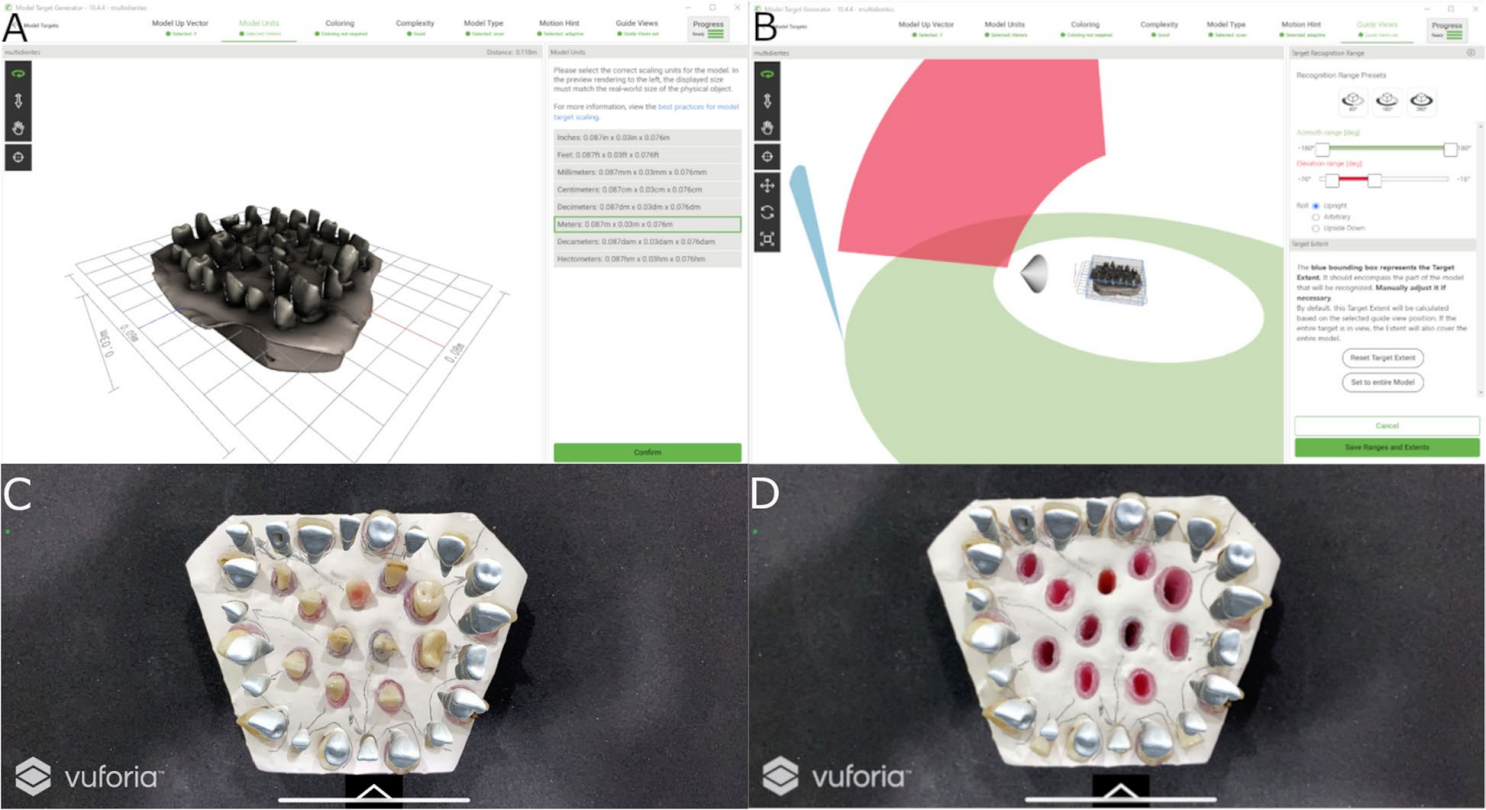
**A**,** B** Planning process in augmented reality device software and **C**, **D** image obtained with the augmented reality appliance with the virtual autotransplanted teeth (white teeth)

On the other hand, the drilling procedure of the osteotomy site of the teeth randomly assigned to the autotransplanted tooth using conventional FT study group was performed completely manually. Indeed, the operator was allowed access to the CBCT and preoperative planning to determine the characteristics of the drilling. Subsequently, the teeth placed inside of the experimental models of epoxy resin were extracted and placed between the teeth placed outside of the experimental model until it adjusted to the previously autotransplanted planned position (Fig. 4). A single operator with 10 years of surgical experience performed all autotransplanted teeth procedures.

A diamond bur with a diameter of 1.2 mm on the active part, a total length of 14 mm, and a working length of 11 mm was used (Ref. 882 314 012, Komet Medical, Lemgo, Germany).

### Measurement procedure

**Fig. 5:**
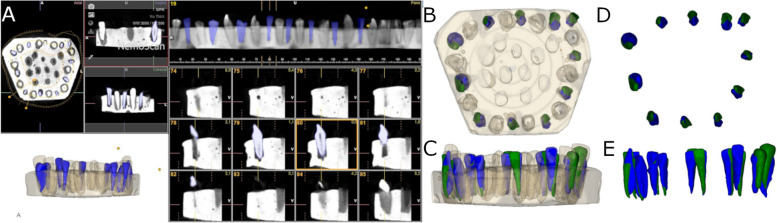
**A** Segmented teeth on the postoperative CBCT scan (blue teeth), **B** apical and **C** lateral view of the planned (green teeth) and performed (blue teeth) autotransplanted teeth with the model of the augmented reality technique, **D** apical and **E** lateral view of the planned (green teeth) and performed (blue teeth) autotransplanted teeth without the model of the augmented reality technique

After performing the osteotomy site preparation and placing the autotransplanted teeth of both study groups, a postoperative CBCT scan (WhiteFox) of the experimental models were taken with the same, previously described exposure parameters. STL digital files from the planning and datasets from postoperative CBCT scans of the two study groups were uploaded to the 3D implant planning software (NemoScan®) and aligned with the best fit algorithm using the 3D implant planning software (NemoScan®). The measurement procedure was performed in the 3D implant planning software (NemoScan®), which automatically measured the deviation between the postoperative location and the virtually planned position of the teeth. Afterwards, the deviation in the most coronal portion (incisal border) and the most apical portion of the access cavity preparations (apex) were calculated in decimals of millimeter (0.01 mm), whereas the overall angle deviation was measured in decimals of degrees (0.01°) (Fig. 5), after marking the incisal border and apex of both planned and performed position of the teeth with a tool of the 3D implant planning software (NemoScan®) by an unique independent observer.

### Statistical tests

All the variables of interest were recorded for statistical analysis with SAS v9.4 (SAS Institute Inc., Cary, NC, USA). Descriptive statistical analysis was expressed as means and standard deviations (SDs) for quantitative variables. Comparative analysis was performed by comparing the mean deviation between planned and performed autotransplanted tooth using Student’s *t*-test, since variables had normal distribution, or Mann–Whitney non-parametric test; *p* < 0.05 was considered statistically significant.

## Results

The results of the reliability of an augmented reality drilling approach and a freehand drilling technique for the autotransplantation of single-rooted teeth were expressed as means, medians, standard deviation (SD), minimum and maximum values in Table 1. Coronal, apical and angular measurements were presented.

**Table 1:** Descriptive statistics of the reliability of AR and FT for the autotransplantation of single-rooted teeth. Measurements at coronal and apical deviations were expressed in millimetres (mm) and angular measurements were expressed in grades (°)

Study group	Location	*n*	Mean	Median	SD	Minimum	Maximum
AR	Coronal	10	2.72	2.45^a^	1.16	1.10	4.20
	Apical	10	2.20	1.95^a^	1.00	1.20º	4.40
	Angular	10	6.63	5.75^a^	3.67	2.20	12.40
FT	Coronal	10	4.62	4.20 ^a^	1.85	2.00	7.70
	Apical	10	4.36	3.90 ^b^	1.99	2.20	8.10
	Angular	10	7.61	7.10 ^a^	4.53	2.30	15.80

^a,b^ Statistically significant differences between groups (*p* < 0.05)

*AR* augmented reality technique, *FT* free-hand technique

**Fig. 6:**
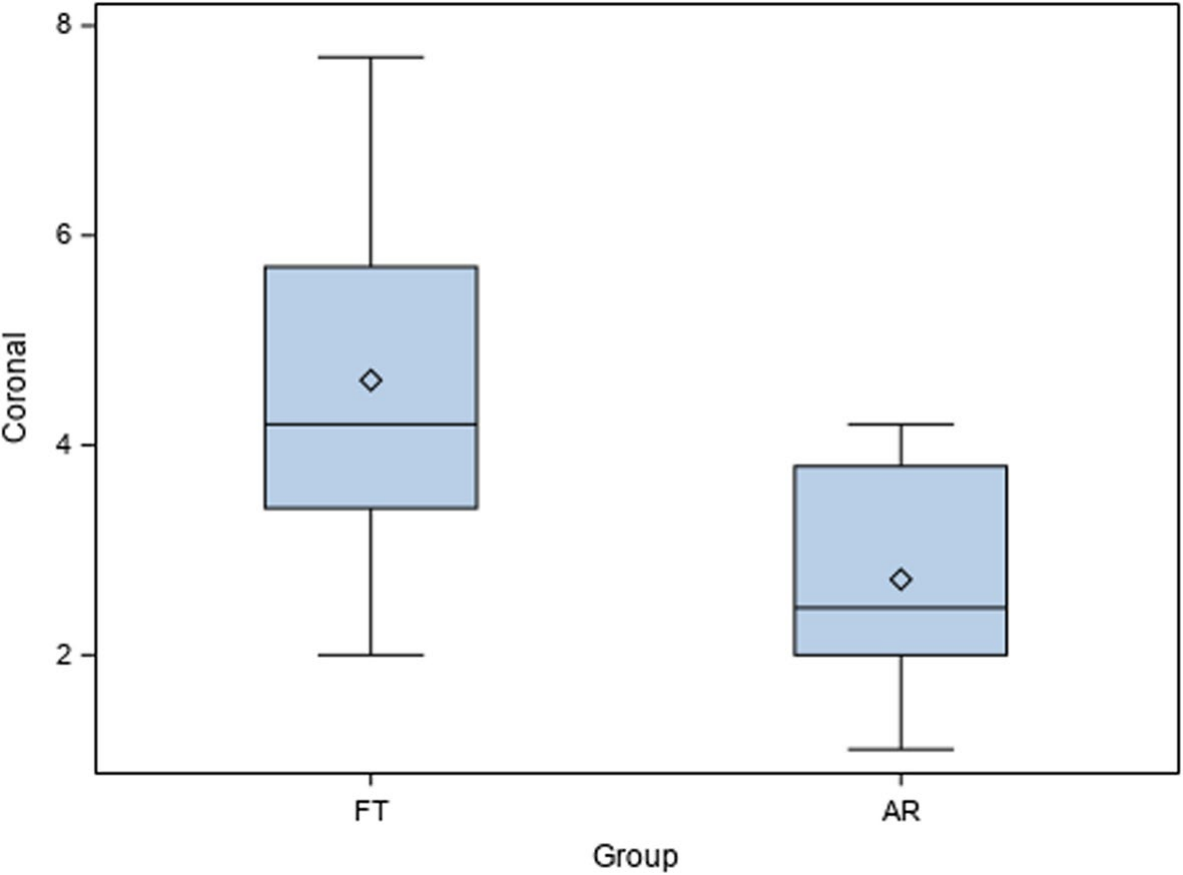
Box plot of the coronal deviations of AR and FT surgical techniques for drilling guidance of the artificial sockets for the autotransplanted teeth. Median is expressed by a horizontal line in each box and mean was expressed by symbol “◊”

Mean comparison between the coronal deviations of AR (2.72 ± 1.16 mm) and FT (4.62 ± 1.85 mm) surgical techniques for drilling guidance of the artificial sockets for the autotransplanted teeth did not show statistically significant differences (*p* = 0.123) (Fig. 6).

**Fig. 7:**
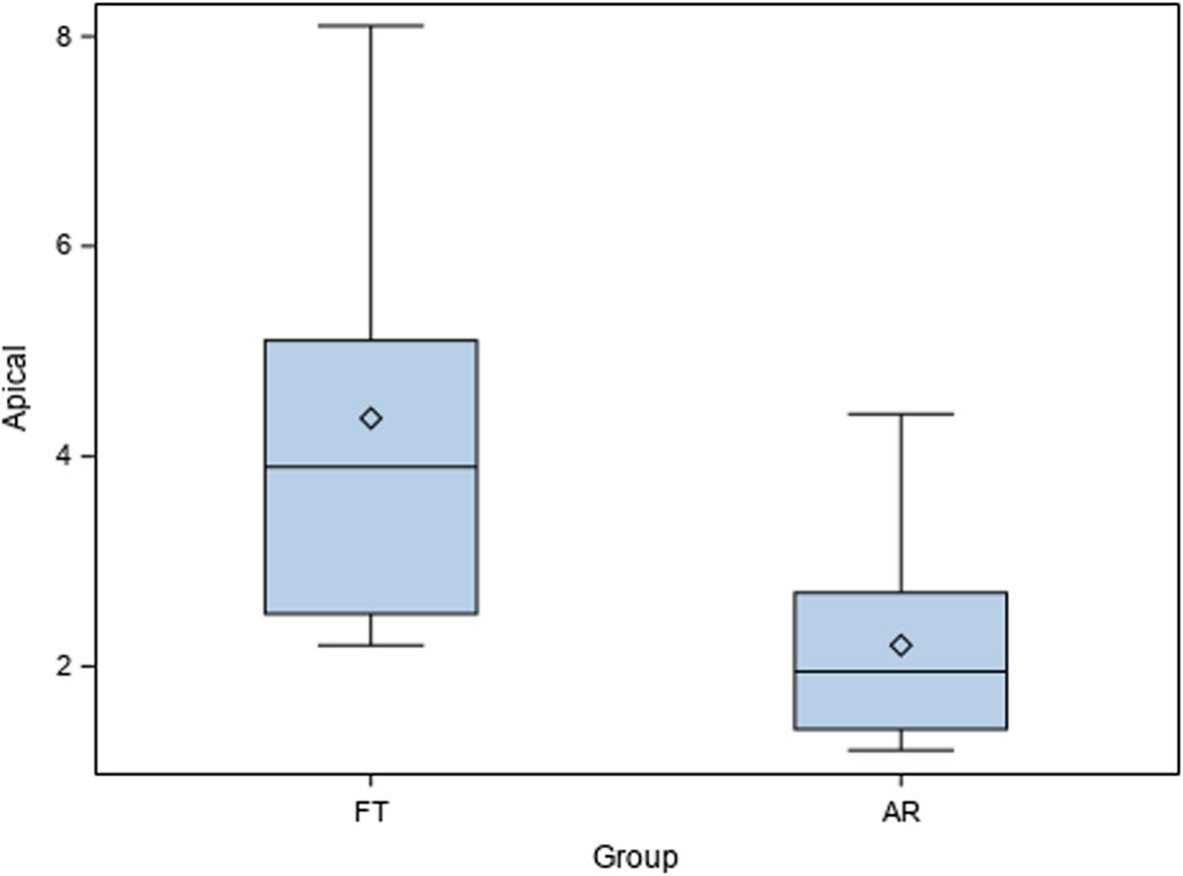
Box plot of the apical deviations of AR and FT surgical techniques for drilling guidance of the artificial sockets for the autotransplanted teeth. Median is expressed by a horizontal line in each box and mean was expressed by symbol “◊”

Mean comparison between the apical deviations of AR (2.20 ± 1.00 mm) and FT (4.36 ± 1.99 mm) surgical techniques for drilling guidance of the artificial sockets for the autotransplanted teeth showed statistically significant differences (*p* = 0.008) (Fig. 7).

**Fig. 8:**
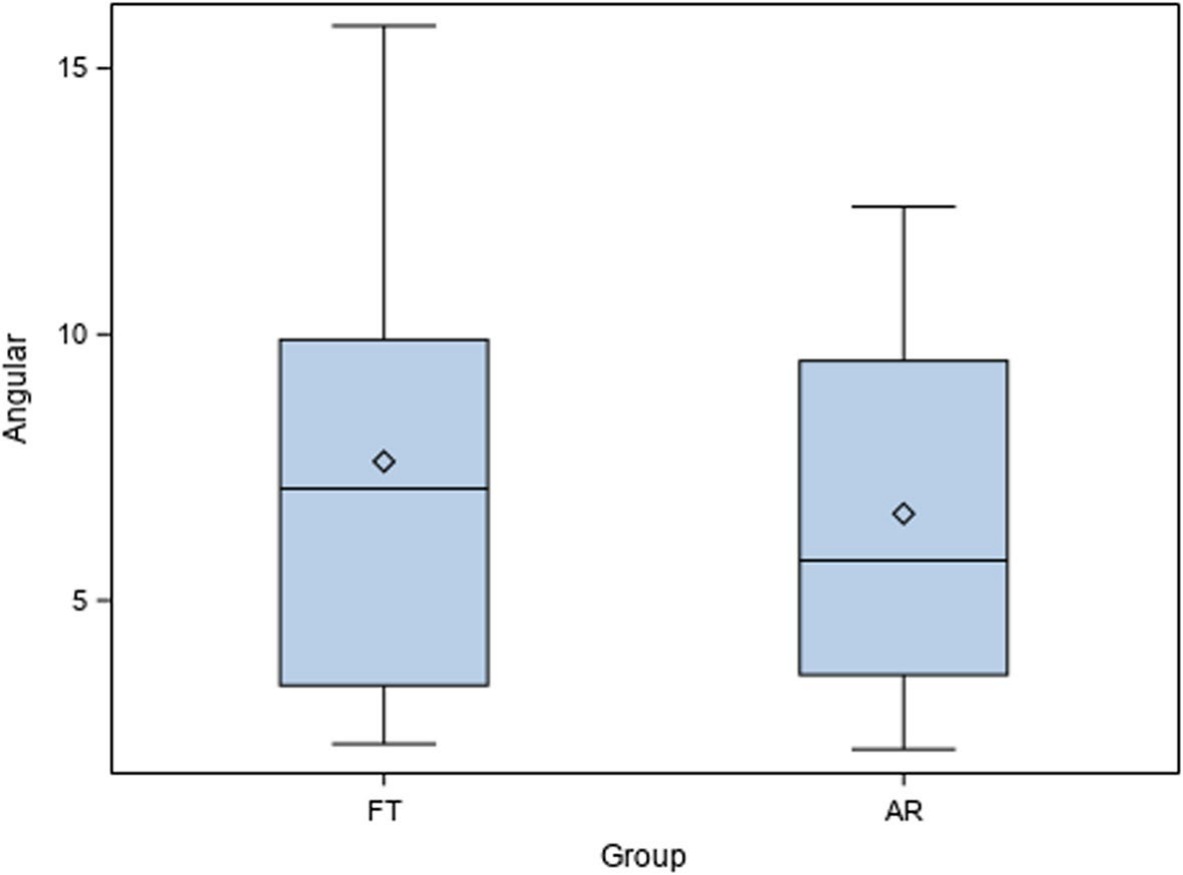
Box plot of the angular deviations of AR and FT surgical techniques for drilling guidance of the artificial sockets for the autotransplanted teeth. Median is expressed by a horizontal line in each box and mean was expressed by symbol “◊”

Mean comparison between the coronal deviations of AR (6.63 ± 3.67 mm) and FT (7.61 ± 4.53 mm) surgical techniques for drilling guidance of the artificial sockets for the autotransplanted teeth did not show statistically significant differences (*p* = 0.340) (Fig. 8).

## Discussion

The results of the present study reject the null hypothesis (H_0_), which postulates that the AR and FT surgical techniques for drilling guidance of the artificial sockets did not show differences for the autotransplantation of single-rooted teeth.

The augmented reality allows blending tridimensional data with the vision of the reality [16, 17]. This computer-aided method brings the clinician a better perception of the patient environment. It is made up by a device worn in the head and on the eyes. It has never been used before in the surgical procedure of autotransplantation. However, it has been studied in other field of dentistry such us for apicectomies [16] and tooth carving [17]. Also, AR can be based on an application of the smartphone which allows superimpose the 3D images on reality and shows image marks into the camera viewer. The AR has proven to be an accessible and potential tool for the improvement of dental practise, but it requires some learning [17, 18]. AR allows maintain the orientation in the reality and it has been previously used with holographic glasses. It can project the 3D images like holograms in the reality in front of the user and the images rotate and can be shown in different angles. This can be used to visualize the root of the tooth and pulp canals in the exact position which cannot be seen directly into the reality [19]. In order to use AR, it is needed a previous CBCT and processed it in a specific software that integrate the 3D images and that scan the reality integrating both. Also, CBCT is needed to obtain 3D-printed tooth or 3D-printed guides [18, 19].

Moreover, Riad Deglow et al. assesed the capability of a computer-aided implant system through surgical templates to perform the socket for tooth autotransplantation, comparing with a manual method by free-hand technique, and reported statistically significant differences at apical deviation; however the authors did not show statistically significant differences (*p* = 0.038) at coronal (*p* = 0.079) and angular (*p* = 0.208) level [9]. This results are aligned with those obtained in the present study.

On one hand, the use of AR has been previously studied in order to localize pulp canals and perform endodontic access [19]. Faus-Matoses et al. showed that AR was more predictable and accurate that free-hand technique doing endodontic access. AR obtained a coronal deviation of 0.76 mm, apical deviation of 0.79 mm and angular deviation of 3.05º. However, manual navigation obtained deviation of 2.77 mm, 2.98 mm and 5.97º respectively [20]. Zubizarreta-Macho et al. compared static navigation system with dynamic navigation system and manual navigation. The authors obtained deviations of 7.44 mm in coronal position, 7.13 mm in apical position and 10.04º in angulation with static navigation system; 3.14 mm, 2.48 mm and 5.58º respectively with dynamic navigation system and 4.03 mm, 2.43 mm and 14.95º respectively with manual navigation [9]. AR seems to be the more precise method for canal root access [9, 20]. AR has also been used for the placement of dental implants, in oncologic patients for the resection of tumours and in apicectomies [21]. Bosshard et al. compared AR with splint-guided apicectomy and did not observe statistical differences in angular deviation (5.33º and 5,23º respectively). Whereas AR obtained less depth deviation than splint-guided apicectomies (0,27 mm and 0,90 mm respectively), neither statistical difference was shown between both techniques [22]. In the present study the AR showed better accuracy in the root apex location of the autotransplanted tooth than the free-hand technique. In the coronal third the mean deviation was 2.72 mm with AR and 4.62 mm with FT. Also, in the apical third the mean deviation was 2.20 mm with AR and 4.36 mm with FT. For angular deviation the means data obtained were 6.63º and 7.61º respectively. Whereas only the apex location has statistical differences between the procedures, with AR the threes variables analysed tend to be decreased.

In the other hand, CBCT has been used for doing autrotranplantation of teeth. Tooth replicas printed from the 3D files allows minimizing extra oral dry time of the donor tooth. This can be achieved because the replica developed by technology in used for shaping the recipient socket for the perfect fitting of the autotranplated tooth. Also, when the tooth fits at first time it is introduced into the shocked the ligament trauma is reduced, and the final position of the tooth will be adapted to the donor alveolar ridge shape. It is important to ensure the accuracy of replica comparing to the CBCT planification. Ker Lee et al. studied the accuracy and reproducibility of printed replicas. It has been shown that the accuracy of the replicas is normally better than 0.5 mm. Commonly, the maximum of the variations of the replica are located on root apex and the replicas were larger than the donor tooth [23]. Moreover, there are different processes for the replica fabrication such us plastic, jetted photopolymer, digital light processing, or 3D printing replicas. The digital light processing and jetted photopolymer replicas showed less differences with the original than the 3D printed replicas [24]. However, 3 printed replicas for the autotranplantation treatment improves the prognosis and predictability of the procedure. Although, autotransplantation can have complications such us the loss of periodontal insertion, root resorption or ankylosis. Also, the autotransplanted tooth normally need endodontic treatment after its transplantation. The pulp vitality is more commonly to be maintained when the patient is between 13 to 20 years old compared to older patients [25]. Autotransplantation of immature teeth has also been reported and has showed good success rate in 20 months of follow up [26]. Given the incompatibility of placing implants in growing patients because of the continuous jaw grow, it is interested to improve the autotransplantation procedure. However, the biomechanics of autotransplanted teeth are not clear and excessive occlusal forces can lead the failure of the treatment. Lahoud et al. studied the influence of occlusal morphology and root shape in the success of autotransplantation. For that, the authors used the model order reduction to analyse the different forces that would be applied to the autotransplanted tooth. They showed that low-intensity axial and lateral forces induces high stress in the cervical part of the root, and this can produce fractures or resorption in the autotransplanted tooth [27]. In immature teeth that were autotransplanted the regenerative endodontic treatment is also an option for maintain the pulp vitality. The pulp of immature teeth can undergo a sterile necrosis due to the replacement of the tooth and the healing involves the introduction of cementum and ligament cells into the tooth apex. This can be achieved by revascularization treatment than is normally indicated one month after the autotransplantarion. The stem cells of the apical papilla are the precursors of the root growth and cell differentiation. The revascularitation can be observed after 6 weeks after the treatment [28]. It is important to maintain the ligament intact and minimizing the extra oral dry time while the extraction of donor tooth is being done [23, 28].

However, computer implant system through surgical templates have shown limitations assigned to each workflow step. Specifically, Unsal et al. highlighted susceptible errors in the CAD/CAM procedures during [29] and Orentlicher [30] and Soardi [31] reported that the total inaccuracy may be due to the sum of mistakes. Moreover, the learning curve associated to all digital processes may affect the accurcay of the dental implant placement [30, 31]. Thus, the authors encourage to increase the knowledge digital workflow processes to reduce the influence on the prognosis treatments.

Moreover, the strength of this study is the application of the augmented reality drilling approach for the autotransplantation of single-rooted teeth. Furthermore, the use of extracted natural teeth allows their extrapolation to the clinical situation.

The promising results derived from this study will lead to new studies analyzing the application of the augmented reality drilling approach for the autotransplantation of multi-rooted teeth.

## Conclusions

Within the limitations of this in vitro study, the results show that the augmented reality appliance provides higher accuracy in the positioning of single-root autotransplanted teeth compared to the conventional free-hand technique; specifically, at apical level.

## Data Availability

Data available on request due to restrictions, e.g., privacy or ethical (Álvaro Zubizarreta-Macho; amacho@uax.es).
